# Dog Bite Prevention: Effect of a Short Educational Intervention for Preschool Children

**DOI:** 10.1371/journal.pone.0134319

**Published:** 2015-08-19

**Authors:** Nelly Lakestani, Morag L. Donaldson

**Affiliations:** 1 Escuela de Medicina Veterinaria, Facultad de Ecología y Recursos Naturales, Universidad Andrés Bello, Santiago, Chile; 2 School of Philosophy, Psychology and Language Sciences, University of Edinburgh, Edinburgh, United Kingdom; University of Tuebingen Medical School, GERMANY

## Abstract

This study aimed to investigate whether preschool children can learn how to interpret dogs’ behaviours, with the purpose of helping avoid dog bites. Three- to five-year-old children (N = 70) were tested on their ability to answer questions about dogs’ emotional states before and after participating in either an educational intervention about dog behaviour (intervention group) or an activity about wild animals (control group). Children who had received training about dog behaviour (intervention group) were significantly better at judging the dogs’ emotional states after the intervention compared to before. The frequency with which they referred to relevant behaviours in justifying their judgements also increased significantly. In contrast, the control group’s performance did not differ significantly between the two testing times. These results indicate that preschool children can be taught how to correctly interpret dogs’ behaviours. This implies that incorporating such training into prevention programmes may contribute to reducing dog bite incidents.

## Introduction

Having a pet dog has numerous physiological and psychological benefits, particularly for children [1–3]. For example, contact with companion animals has been suggested to enhance the development of children’s self-esteem [3]. Unfortunately, injuries caused by dog bites are a problem amongst children. Statistics show that there are more than 4.7 million dog bites per year in the US [4]. There is no data reporting the numbers in the whole of Europe but studies suggest that up to 10% of the population has suffered from dog bites in their lifetime [5]. Numerous studies have shown that children are more at risk of being bitten than adults, and that most victims are bitten by a familiar dog [6–17]. Children may be expected to suffer from more serious injuries than adults, and they may therefore be over-represented in studies based on hospital admission. But, studies based on telephone surveys indicate that children are more likely to be bitten than adults [6, 7, 14, 18], suggesting that the gravity of injuries is not the only reason why children are reported to be more at risk of suffering from dog bites. Since owning a pet dog has many benefits, it is important that children have the opportunity to do so. Therefore, solutions that allow children to interact safely with dogs must be found.

There have been a number of dog bite prevention programs created but only four have been scientifically tested (Table 1). Two of the programmes tested were aimed at primary school children [19, 20] and two at preschool children [21–23]. Like most prevention programmes, there should be different programs for different age groups. In the case of dog bite prevention it is particularly important to focus on preschool children since younger children seem to be more at risk of being bitten than older children [14, 24]. The reasons why children are more likely to be bitten than adults are still to be clarified, but studies suggest that it is caused by the way children interact with dogs [17, 25]. Children below the age of seven initiate interactions with dogs more often than older children, mostly to pet, hug or kiss the dog. In addition, children below the age of six are significantly more likely to misinterpret dog behaviour than older children [25]. We may therefore infer that many dog bite accidents happen because younger children unintentionally provoke the dog, probably because they do not know how to interact with it. This, in turn, may be because younger children do not know how to interpret dog behaviour, or how to recognise potentially dangerous situations.

**Table 1:** Summary of studies evaluating dog bite prevention programme for children.

Name of programme evaluated and reference	Age of participants	Nature of intervention	Measures	Outcome [& comments]
BARK (Be Aware, Responsible, and Kind) Dog Bite Prevention Program [19]	7- to 9-year-olds	School-based, 60 minute interactive lesson including workbook, video and role-playing with life-size toy dogs. Supplemental take-home materials for children and parents. Aimed to increase understanding of dog behaviour and body language and how to behave in particular situations to prevent dog bite incidents.	Responses to questionnaire (administered 2 weeks after intervention) about various scenarios involving dogs—judgements about how child would behave in particular situations and about whether particular situations are safe.	Correct responses to questionnaire were more frequent after than before intervention. This included a significant increase in identifying which dog was scared on basis of dog’s body language. [No control group. Only one question assessed understanding of dog’s body language. Does not assess whether children can apply learning to situation with real dog.]
Prevent-a-Bite [20]	7- to 8-year-olds	Lesson (30 minutes) with dog-handler and real dog—demonstrating how to recognise dog states (e.g. friendly, angry, frightened) and how to behave safely around dogs, (e.g., how to approach dogs)	Behavioural observations, seven to ten days after training, comparing children who had received training to those who had not, when presented with the opportunity to approach an unfamiliar dog.	Children who had received the intervention displayed greater precautionary behaviour than children in the control schools (who had not received any intervention). [Does not assess understanding of dog’s body language. Does not assess preschool children.]
Delta DogSafe [23]	4- to 5-year-olds	30 minutes programme, using photographs to educate children about how to behave if confronted by a dog and how to identify risks, such as a dog sleeping, as well as how to interpret the dog’s body language.	Learning was assess through photograph based tests, showing dogs in different high and low risk situations, aimed at assessing if children recognised situations as safe or unsafe. The children were tested before the intervention and four weeks after the intervention.	The dog safety program resulted in a significant increase in the ability of children to identify high-risk situations for up to eight weeks in all three experimental groups. The benefits were highest for those children who had received training and whose parents were also given information. [Only photograph based tests, no videos or live dogs. Questions only examined if they could identify dangerous situations, and not whether they had learned how to interpret dog behaviour.]
The Blue dog [22]	3.5- to 6-year-olds	Software programme depicting different situations in which a dog may bite, through stylized drawings of a blue coloured dog. Supplemented by a guide book that teaches parents about dog-child interactions and canine aggression. The software programme teaches children to recognize situations in which a dog may bite by having them decide whether the cartoon child in the software should interact with the dog or undertake another activity.	Children completed 3 tasks to evaluate dog safety pre- and post- intervention: (a) pictures (recognition of safe/risky behavior), (b) dollhouse (recall of safe behavior via simulated dollhouse scenarios), and (c) live dog (actual behavior with unfamiliar live dog).	Children using Blue Dog had greater change in recognition of risky dog situations than children learning fire safety. However using the Blue Dog programme did not result in an increase in safe behaviour with real dogs nor when simulating situations with a doll’s house. [Only examined if they could identify dangerous situations, and not whether they had learned how to interpret dog behaviour.]
The Blue dog [21]	3- to 4- year-olds		Measure of children’s responses to potentially unsafe situations with dogs, pre and post use of the Blue Dog Software.	Children's knowledge significantly increased after 12–20 minutes of using The Blue Dog software, and knowledge was retained for up to two weeks. [Only examined if they could identify dangerous situations, and not whether they had learned how to interpret dog behaviour.]

The first dog bite prevention program tested was the BARK (Be Aware, Responsible, and Kind) Dog Bite Prevention Program [19]. See Table 1 for details of the study. The program appeared to be highly effective in helping children understand how to prevent or avoid potentially threatening situations involving dogs. However, the program was found to be most effective for older participants which may cause problems since younger children seem to be more at risk than older children [14, 24]. Moreover, the results of this study do not give information on whether the children will actually be able to apply what they have learned when faced with a real dog. A study carried out in Australia addressed the latter problem by exposing the participants to a real dog [20]. The study investigated the effectiveness of a dog bite prevention programme for seven to eight year old children by observing the number of children who breached proscribed behaviours (for example, patting the dog incorrectly), Table 1. This prevention program was effective and showed that children are able to use what they learn during the prevention program to interact with a real dog. However, this study was also only aimed at primary school children. Three studies have evaluated prevention programs aimed at preschool children. A study by Wilson, Dwyer, & Bennet [23] investigated parents’ beliefs about their children’s behaviour around familiar and strange dogs, and evaluated the impact of a brief educational dog safety program on kindergarten children. The children who had participated in the training learned how to identify dangerous situations from role-playing and pictures, and they were able to use the information to recognise dangerous situations illustrated by pictures that they had not seen during the training session. However, even though the children were taught how to interpret dog behaviour, the questions they were asked only examined if they could identify dangerous situations, and not whether they had learned how to interpret dog behaviour. More recently two studies evaluated an interactive software programme aimed at preschool children called The Blue Dog [21, 22]. These studies focused on children's ability to recognize situations in which a dog may bite based on the scenes presented in The Blue Dog software. One study [21] demonstrated that children's knowledge significantly increased after 12–20 minutes of using The Blue Dog software, and that knowledge was retained for up to two weeks. The other study [21] aimed to evaluate whether after using the software children would change their behaviour with real live dogs and whether their knowledge would be transmitted to other situations, such as simulating dangerous situations with a doll’s house. This study also showed that children's knowledge of safe situations in the software significantly increased. However using the programme did not result in an increase in safe behaviour with real dogs nor when simulating situations with a doll’s house. Perhaps if Schwebel et al.’s [21] study had used videos of real dogs to teach children about dog behaviour, it would have increased children’s safe behaviours around a real dog. The Prevent-a-Bite programme for primary school children [20], where the training involved real dogs, resulted in safer behaviour around a real dog. Using videos during training may also be efficient, whilst more easily implemented than using a real dog.

The examples given above of programs with preschool children used still images or stylised drawings of dogs and not videos of real dogs. It has been suggested that video, rather than drawings, may be a better medium for raising children’s awareness of hazardous situations, since they may be more memorable/meaningful to young children in particular [26]. Moreover, they investigated whether children could learn how to recognise dangerous situations rather than whether they could learn how to make judgements about dogs’ behaviour. The aim of a prevention program is to teach children how to behave in different situations and it is important to give them the necessary background information to ensure they remember. Therefore, if children are capable of learning how to observe and make judgments about dog behaviour, they may be more likely to remember how to behave in different situations. In other words, remembering what a behaviour means may help children to remember how to interact with the animal.

The aim of the present experiment is to investigate whether children as young as four years of age are (a) able to learn how to interpret the behaviours of dogs (b) whether they can generalise what they have learned to different dogs, (c) use videos of real dog as a teaching tool. As far as we are aware, this study is the first to address these questions. In the light of previous dog bite and other injury prevention programmes (e.g. [23]), it seems likely that preschool children will demonstrate some ability to learn how to interpret dog behaviour after a short educational session, but the extent and nature of this learning needs to be explored. The present study will therefore investigate whether children are able to interpret the behavioural states of dogs other than the dogs presented to them during the intervention. Learning may be shown not only in improved performance on the recognition of the dogs’ state, but also in increased ability to justify answers by referring to those behaviours/features that provide relevant cues to the dogs’ emotional states and that were therefore highlighted during the educational session. In addition, performance will be compared between children who either own or do not own dogs. Children who own dogs may be more sensitive to learning about dog behaviour and may perform better than children who do not own dogs. Or at a more general level, children who own pets may be more sensitive to animals’ behavioural cues than children who do not own pets. Videos of dogs will be used as the training materials because behaviours such as fear, aggression and friendliness are composed of movements and sounds, which cannot be represented in pictures or by puppets. If the intervention were to be successful, videos would be an ideal tool to be incorporated into a prevention programme that could be used by the public.

## Method

### Participants

The participants were 70 nursery school children, between three and five years old (mean age: 4.4 years, SD = 0.3). The participants attended one of five nursery schools, all of which were in the city of Edinburgh. The children were randomly assigned to the intervention group (36 children, mean age = 4.3, SD = 0.38) where they were taught about dog behaviour or to the control group (34 children, mean age = 4.4, SD = 0.33) where they were taught about wild animals. In each school, half of the children were assigned to the control group and the other half to the intervention group, and children from the same section (sharing the same classroom) were always assigned to the same condition (control or intervention).

Parents of all the children who participated were provided with informed written consent form prior to the pre-test. The form also included questions on whether they had a pet in the family and whether the child had been bitten by a dog in the past.

### Design

The children in both groups participated in three experimental sessions: (i) a pre-test, (ii) an educational intervention for the intervention group or a control activity for the control group, (iii) a post-test. All three sessions took place in the nursery. The educational intervention involved watching a video about dog behaviour, whereas the control activity involved watching a video of the same length about wild animals. In both conditions, the videos were presented to groups of three to five children (median four children). For the pre-test and post-test, each child was shown the videos individually on a computer in a quiet room, in the nursery. The video clips were shown in a different random sequence for each child. The children were asked the questions verbally and their answers for each video were written down.

### Materials

The pre- and post-tests involved showing the children 14 short video clips (6–10 seconds, shown in random order) of dogs performing different behaviours: five friendly dog clips, three defensive aggressive (aggressive because of fear) dog clips and six fearful dog clips (see Table 2). The length of each clip was determined by the time taken by the dog to perform the behaviour. Out of the 14 video clips used in the pre- and post-tests, six were also used during the educational sessions (“training videos”, Table 2). The aim was to investigate if the children were able to apply what they learned with the six dogs during the intervention in order to interpret the states of the other eight dogs that were used only in the pre- and post-tests (“test only videos”).

**Table 2:** Characteristics of each of the 14 videos (each row corresponds to one video).

State of dog	Dog breed	When video used
Friendly	Black Labrador	tests and training
	Husky	tests and training
	Weimaraner	tests only
	Black Labrador	tests only
	Cocker	tests only
Fearful	Schnauzer	tests and training
	Greyhound	test and training
	Pomeranian	tests only
	Ridgeback	tests only
	Boxer	tests only
	Black Labrador	test only
Aggressive	German shepherd	tests and training
	Bedlington cross	tests and training
	Husky	tests only

The 14 clips were selected by presenting a larger set of 16 clips to nine experts. The experts were three professional pet counselors and four veterinary behaviorists who were asked to rate the dogs’ behavioral and emotional states. The ratings were free-response, in that the raters were asked to write down what behavior they thought the dog was displaying, without being given a list of alternatives to choose from. The videos that were rated as ambiguous or for which the raters’ descriptions did not match were excluded, leaving 14 clips on which the raters agreed about the dogs’ emotional state. For the videos of aggressive dogs, most raters reported that the dogs were defensive and/or displayed fear, as well as aggression. We decided to label these as “aggressive” dogs because even though aggression may be displayed in many different contexts and because of different causes, it is often displayed because of fear. The videos were therefore considered to adequately represent aggressive dogs behaving so due to fear.

The educational intervention video, on dog behaviour, was composed of a total of six clips representing each of the following three behavioural states: fearful (2 clips), aggressive (2 clips) and friendly (2 clips). All of the clips were presented in the pre- and post-tests. The intervention video was made up of individual clips so that the instructor had control over the video (e.g. to facilitate pointing at the dogs' body parts) and over the rate at which information was presented.

The control video was made up of six clips, representing elephants, giraffes, tigers, zebras, hedgehogs and kangaroos, in their natural environment, performing a range of normal behaviours. The total length of the video was the same as the educational intervention video.

In contrast to the videos used in the educational intervention, which comprised an equal number of videos for each emotional state, the videos used in the pre- and post-tests comprised unequal numbers of videos for each emotional state, due to difficulties in obtaining videos. Videos of defensive dogs were particularly difficult to obtain, whereas videos of fearful dogs were easiest to obtain. Because of this and because an attempt was made to include dogs with a variety of features (e.g. regarding tail length), the number of test videos for each emotional state varied as shown in Table 2.

### Procedure

Ethical approval was obtained from the Ethics Committee of the School of Philosophy, Psychology and Language Sciences of the University of Edinburgh.

### Pre- and post-tests

At the start of the pre-test, the researcher showed the children three cartoon-style drawings depicting happy, scared and angry facial expressions and asked what they thought they meant, to check that the emotions were recognized correctly. The pre-test was administered two to three days before the educational intervention. Each child was interviewed individually and shown each clip as many times as he/she asked for. Usually the children asked to watch the videos only once. On two occasions, once in the pre-test and once in the post-test, two different children asked to watch a video three times. After each clip, the child was asked: “How is this dog feeling? Is it happy, scared or angry?” They were presented with a piece of paper with the three cartoon-style drawings of a happy, scared and angry face that they could refer to in order to answer the question. They were then asked: “How do you know it’s feeling that way?” The children were shown the same video clips following the same protocol two to three days after being shown the educational videos (post-test).

### Educational intervention

The children in the intervention group were shown the dog behaviour training videos. At the start of each clip, the trainer introduced it by referring to the dog’s behavioural state (angry, scared, happy). The children were then asked: “How do you know it is feeling that way?” and when necessary their answers were corrected or elaborated by using the comments shown in Table 3. Each clip was played twice and after showing the clips following this protocol a first time, there was a short pause of a few minutes during which the children were free to talk about whatever they wished. After the pause, the clips were played again one time each and after each clip, the children were asked to say how the dog was feeling and how they knew it was feeling that way. The answers were corrected when necessary. Every effort was made to encourage all the children to participate and comment on the clips.

**Table 3:** Information given to the children during the training video.

Dog state	Dog breed	Comments given during video
Friendly	Black Labrador	“This dog is happy. The tail of the dog is wagging and the dog is smelling and saying hello to another dog.”
	Husky	“This dog is happy. The tail of the dog is wagging and the dog is smelling and saying hello to the person.”
Fearful	Schnauzer	“This dog is scared. The dog is shaking, and not moving very much. Also his tongue is out, so when the tongue of the dog is out it doesn’t always mean that the dog is happy. “
	Greyhound	“This dog is scared. The dog is not moving very much, his ears are down and flat against his head (imitation of the position of the ears with hands on the head and ask the children to do it as well). Also the tail of the dog is between his legs.”
Aggressive	German Shepherd	“This dog is angry. The dog is barking and pulling on the lead. Also it is wagging its tail but it doesn’t mean it is happy. Sometimes dogs get angry when they are scared.”
	Bedlington cross	“This dog is angry. The dog is barking and walking moving backwards towards the person so it shows that he is also a little bit scared”.

At the end of the study (after the post-test) a short talk was given to all children in order to inform them about how to behave in the presence of a scared dog and particularly to make them aware not to approach one. The children were asked how they would behave in the presence of a scared dog, and were given advice according to their answer.

### Control activity

The children in the control group were shown a video about wild animals and were asked to identify the animals in response to the question “Can you tell me what animal this is?” They were also asked: “How do you know it’s (e.g. an elephant)?”, “Where do (e.g. elephants) live?”, “What do (e.g. elephants) eat”? Each clip was played twice and after showing the clips following this protocol a first time, there was a short pause of a few minutes during which the children were free to talk about what they wished. After the pause, the clips were played again one time each and after each clip, the children were asked what the animals were, how they knew which animal it was, where the animal lived and what the animal ate.

### Data analysis

The total number of correct answers was calculated for each child for the pre-test and the post-test as follows. For the friendly dog videos, only the answer “happy” was counted as correct, and for the fearful dog videos only the answer “scared”. For the aggressive dog videos, the answers “angry” and “scared” were both coded as correct, because the dogs were displaying aggression as a result of fear. Therefore, even though the prevalent behaviour of the dog was aggression and the most obvious answer was “angry”, the answer “scared” was also correct. In fact, the children were taught during the training that dogs could get angry because they were scared.

The children’s performance for the “training videos” was compared to their performance for the “test only videos” by comparing the number of correct answers the children in each group (control/intervention) gave in the pre- and post-tests for each type of video.

The number of times each child reported a feature/behaviour of the dog as a response to the question “How do you know it is feeling that way?” was counted. The behaviours were grouped into “targeted” and "untargeted” behaviours. The targeted behaviours corresponded to the behaviours that were pointed out to the children when they were taught how to interpret the dogs’ behaviour (Table 3). For fearful dogs, these behaviours corresponded to “stand still/ quiet”, “tail (down)”, “shiver”, “pant” and “ears (down)” because these represent features that are useful for recognising fear in dogs. “Back up”, “bark”, “pull lead” and “tail (wag and up)” are features and behaviours that are associated with aggressive behaviour. “Say hello” and “tail (wag and up)” are associated with friendly behaviour. The untargeted behaviours were all the other behaviours that the children used to describe the videos and which were not taught to them during the intervention. These were: jump/play, hide/stay close to owner, walk around, run, whine, wanting to get out of the room, smile, head down, looking around, biting, showing teeth, and other behaviours that were reported on fewer occasions.

The total number of features reported per child was also analysed as a function of training. The total number of features each child reported in the pre-test and the post-test was compared for children in the intervention and control groups.

ANOVA and paired t-tests were used when the data met the parametric requirements. Mann-Whitney U tests and Wilcoxon Signed Ranks tests were used on the non-parametric data.

## Results

### Effect of the educational session for children the intervention and control groups

This section reports the findings regarding answers given to the question: “How is this dog feeling?”. Analysis of covariance was used to compare the number of correct answers given in the post-test by children in the control with that of children in the intervention group, using the results of the pre-test as the covariate. The mean number of correct answers given by trained children at the post test was significantly higher than that of children in the control group (*F*(1, 67) = 12.285, p = .001, η2 = 0.2), even when the effect of any differences in pre-test scores was controlled for. Trained children gave on average 7.3 correct answers while children in the control group gave an average of 5.3 (Table 4). Moreover, the number of correct answers given at the pre-test was significantly related to the number of correct answers given at the post-test (*F*(1, 67) = 16.438, p = .000, r = 0.44).

**Table 4:** Mean number of correct answers, mean/median number of targeted behaviours and 95% confidence intervals for children in the different conditions and for the different types of video shown, with results of statistical analyses.

			Number of correct answers			Mean number of targeted behaviours reported			
			Mean	95% CI	Statistical analysis	Mean	Median	95% CI	Statistical analysis
Intervention group n = 36		Pre-test	5.0	4.29–5.76		4.4		3.41–5.37	T-test, T -
		Post-test	7.3	6.5–8.2	ANCOVA F =:	9.1		7.39–10.83	6.413**
Control group n = 34		Pre-test	5.0	4.21–5.73	12.285**	5.1		3.88–6.29	T-test, ns
		Post-test	5.3	4.43–6.22		5.4		4.03–6.74	
Intervention group n = 36	Training	Pre-test	2.2	1.78–2.61	Wilcoxon		3	1.99–3.17	Wilcoxon
	video	Post- test	3.6	3.06–4.1	T = 9.3**		5	4.22–5.77	T = 526.5**
	Test	Pre-test	2.8	2.31–3.35	Wilcoxon		2	1.33–2.28	Wilcoxon
	videos	Post- test	3.8	3.17–4.33	T = 11.4*		3.5	3.01–5.13	T = 402.0**
Control group n = 34	Training	Pre-test	2.6	2.12–3.05	Wilcoxon,		3	2.24–3.63	Wilcoxon,
	video	Post- test	2.6	2.19–3.11	ns		3	2.29–3.70	ns
	Test	Pre test	2.4	1.91–2.85	Wilcoxon,		2	1.51–2.78	Wilcoxon,
	videos	Post test	2.7	2.22–3.14	ns		2	1.63–3.13	ns

** p<0.01,

* p< 0.05, ns: not significant

### Training and test videos

Children in the intervention group had a mean of 2.2 correct answers in the pre-test (*N* = 36) and a mean of 3.6 in the post-test (*N* = 36) for the training videos. For the test only videos, they gave a mean of 2.8 correct answers in the pre-test (*N* = 36) and a mean of 3.8 in the post-test (*N* = 36) (Table 4). The number of correct answers given by children in the intervention group significantly increased from the pre-test to the post-test for both the “training videos” and the “test only videos” (training videos: Wilcoxon *T* = 9.3, *p* < 0.001, test videos: *T* = 11.4, *p* < 0.05). For the control children and for both types of videos, there was no significant difference in the number of correct answers given between the pre-test and the post-test.

### Dog behaviours reported

This section refers to the analysis of the answers to the question “How do you know the dog is feeling that way?”

The number of features reported by the children in the intervention group was significantly lower in the pre-test (*M* = 7.7) than the number of features reported in the post-test (*M* = 11.7, *t* (35) = -4.781, *p* < 0.001). No significant difference was found for children in the control group, M_pre-test_ = 7.7, M_post-test_ = 8.5.

The mean number of targeted behaviours reported by children in the intervention group increased significantly from an average of 4.4 in the pre-test to 9.1 in the post-test (*t* (35) = -6.413, *p* < 0.01). Only the children in the intervention group were taught with the targeted behaviours. To ensure that those were not words that the children would naturally start using after the pre-test the number of targeted behaviours reported by the control children was also analysed for the different tests. The control children’s answers did not differ significantly between the tests (Table 4).

For the children in the intervention group, the number of targeted behaviours reported in the post-test was significantly higher than in the pre-test for both training and test only videos. The mean number of targeted behaviours reported per child was 3 in the pre-test and 5 in the post-test for the training videos (Wilcoxon T = 526.5, *p* < 0.01), and 2 in the pre-test and 3.5 in the post-test for the test only videos (*T* = 402; *p* < 0.01). The number of untargeted behaviours reported did not vary between the pre-test and the post-test. There was no such effect for the control children. Their answers did not vary significantly between the tests (Table 4).

A more detailed analysis of the targeted behaviours category revealed which specific behaviours were reported by the children. Children in the training group reported the following behaviours significantly more in the post-test than in the pre test: stand still (Wilcoxon *T* = 8, *p* < 0.05), tail (*T* = 25, *p* < 0.01), say hello (*T* = 21, *p* < 0.01), shiver (*T* = 15, *p* < 0.001), bark (*T* = 19, *p* < 0.05). Stand still was reported eight times in the pre-test and 29 times in the post-test. Tail was reported 19 times in the pre-test and 87 times in the post-test. Say hello was reported 29 times in the pre-test and 59 in the post-test. Shiver was only reported three times in the pre-test and 17 times in the post-test. Bark was reported 84 times in the pre-test and 107 times in the post-test. In the pre-test, none of the children mentioned the ears but in the post-test “ears” was reported five times by three different children in the intervention group.

There was no significant difference for the children in the control group for any of the comparisons.

### Relationship between correct answers and behaviours reported

This section will examine the relationship between the number of correct answers given to the question “How is the dog feeling?” and the targeted/untargeted dog behaviours/features reported in response to the question “how do you know it is feeling that way?” In order to investigate this relationship, a Spearman’s rho correlation was carried out on all the children’s answers, irrespective of treatment group (intervention/control) and type of test (pre/post tests). The number of correct answers given by the children was positively correlated to the number of targeted behaviours reported (*r*_*s*_ = 0.441, *p* = 0.001) but not correlated to the number of untargeted behaviours (*r*_*s*_ = 0.074). Further correlations were then carried out to investigate whether this relationship between correct answers and reporting targeted behaviours held for both treatment groups and for their performance both in the pre-test and in the post-test. For the intervention group in the post-test, there was a significant positive correlation between the number of correct answers and the number of targeted behaviours reported (*r*_*s*_ = 0.543, *p* < 0.001). This is consistent with the argument that the effectiveness of the training was due (at least partly) to directing children’s attention to relevant features of the dogs’ behaviour. In addition, though, significant positive correlations between these two aspects of performance were found for the intervention group in the pre-test (*r*_*s*_ = 0.441, *p* = 0.013) and for the control group in the post-test (*r*_*s*_ = 0.341, *p* < 0.05). Since these correlations are smaller than the correlation for the intervention group in the post-test, it could still be argued that training enhances attention to relevant features. At the same time, the significant positive correlations for the children who had not (yet) received training supports our assumption that the targeted behaviours were the appropriate behaviours to highlight in the training, since untrained children who gave correct answers tended to report attending to these features even though they had not been taught that these were the ones to look at in order to recognise the dog’s state.

### Other factors

There was no significant difference between the performance of children owning dogs and children owning other pets or not owning a pet. The number of correct answers given based on pet ownership are shown in Table 5. There also was no significant difference between the performances of children from the different schools. None of the children had been bitten by a dog in the past so it was not possible to assess the influence of this factor on learning.

**Table 5:** Mean number of correct answers and standard deviation (SD) by pet ownership status.

		Pre-test		Post-test	
		Mean	SD	Mean	SD
Dog owner	Intervention group (n = 5)	6.60	1.14	6.80	1.92
	Control group (n = 6)	6.33	1.75	6.5	1.64
Other pet owner	Intervention group (n = 15)	6.13	2.26	9.07	2.25
	Control group (n = 7)	5.00	1.53	6.00	2.52
Not a pet owner	Intervention group (n = 16)	6.50	2.34	8.13	2.75
	Control group (n = 21)	5.86	2.37	6.67	2.27

## Discussion

### Effect of educational intervention

The aim of this study was to assess if a short educational intervention would result in increased knowledge about dog behaviour in preschool children. The intervention was successful. Children who had been trained improved in their ability to interpret the state of dogs. Learning was measured by the number of correct answers given when assessing the state of the dogs and by the types of behaviours reported. Trained children gave on average two extra correct answers after the training session. Although this may appear to be quite a small gain, it is worth bearing in mind that the scale of the intervention was relatively modest in that it consisted of only a single 10-minute session.

Another aim of the study was to investigate if preschool children were able to apply what they had learned about recognising the state of one dog to another dog. In Wilson, Dwyer and Bennet’s [23] study children were taught how to behave around dogs by looking at pictures of dogs in a number of situations. The children were able to apply what they had learned and to report the correct behaviour to adopt when shown similar situations but with different dogs from the ones they had seen during the educational intervention. Children in the present study successfully learned how to recognise the state of dogs that they had been taught with during the intervention. And their ability to interpret the state of the dogs that they had not seen during the training session also improved. This suggests that if young children are taught how to recognise the state of a dog they may be capable of using the same cues in order to recognise the state of a different dog. Schewbel et al.’s study [22] carried out with live dogs demonstrated that 3.5- to 6-year-old children who were taught about identifying safe situations involving dogs (but not about dog behaviour) were better at recognising risky situations. However, when presented with a live dog they did not engage in safer behaviour. An important issue to investigate in future research is whether similar results would be obtained regarding children’s ability to interpret dog behaviour when presented with a live dog.

The reason why children had more difficulty in interpreting the state of the dogs that they had not seen during training may not only be due to the fact that they were not able to transfer all they had learned from the dogs that they had seen during the training to other dogs but also to other factors. It could be due to developmental factors, which may make it more difficult for children to generalise during the preoperational stage of their development [27]. It may also be that the dogs in the videos that were used for training were generally easier to interpret because of their behaviour or because of the way they looked. It may not be as easy to interpret friendly behaviour in a dog that has a short tail compared to one that has a long tail. Although an effort was made to have dogs with a variety of features in the training session, it was only possible to use a limited number of dogs and a limited amount of information because of the children’s young age and short attention span. The features of the dogs used during training may have been too different from the ones that were not used during training. This problem could be controlled by investigating if dogs’ features (e.g., coat colour, ear size and tail size) have an effect on people’s interpretation of their behaviour. A training program could then be created by balancing the number of dogs in the training and testing sessions according to the features that influence people’s interpretation of dogs’ behaviour.

Another indication that the children had learned was that trained children used the words the instructor had used in the training session more than children in the control group. The features and the behaviours that were pointed out to the children during the training session (“target behaviours/features”) were chosen because they were considered to be important to attend to in order to correctly interpret the dogs’ state. Therefore the fact that children who had been trained reported more of those behaviours suggests that they were attending to these features and that these helped them in making the correct decision about the dogs’ state. Furthermore, the results showed that the children who were giving correct answers in the pre-test reported looking at the behaviours necessary to interpret the dogs’ state correctly (“target behaviours/features”). It can be deduced from this that giving correct answers was a result of attending to the appropriate features of the dogs.

### Limitations of intervention

A number of issues were not investigated and should be addressed in future research. These issues are highlighted by Chapman et al.’s study [20], where children’s learning was tested in the presence of an unfamiliar real dog. The first issue is whether preschool children are capable of applying what they have learned about dog behaviour to the way they behave towards a real dog. Ideally, children would need to be tested when in the presence of a live dog. Such an experiment might be difficult to run in the United Kingdom due to difficulties with obtaining ethical consent, however it may be possible to do since previous studies have been carried out with live dogs in the United States and Australia. The second issue which was not investigated in the present study, and has not yet been investigated in other studies, is whether children will behave similarly when presented with a familiar and an unfamiliar dog. This is an important question since the majority of dog bite injuries result from familiar dogs [16]. Last, but not least, so far no studies have succeeded in directly testing whether teaching children about dog behaviour, and/or safe behaviour around dogs, results in a reduced number of dog bites. Investigating this issue is difficult because it requires a longitudinal study, whereby families with young children would be given the opportunity to be taught about preventing dog bite incidents and would be followed up over a number of year to assess whether they are less likely to be bitten by dogs compared to the general population.

### Contribution to future dog bite prevention programs

With 31% and 36% of households owning dogs in the UK and the USA respectively [28, 29] and considering that children are mostly bitten by dogs that they are familiar with [6], the importance of educating children on how to prevent dog bite incidents should not be under-estimated. None of the prevention programmes evaluated have been shown to be completely effective in preventing dog bite incidents [16, 19–23]. These previous studies have, however, shown that preschool children can learn about circumstances in which it is safe or not safe to approach a dog. The present intervention shows that children are able to learn how to recognise the state/emotions of dogs and it was effective in directing the children’s attention to the appropriate behaviours/features of the dogs. Since preschool children seem to be able to learn about dog behaviour, there is clear potential for enhancing prevention programs by not only teaching children how to behave in different circumstances but also teaching them how to use the behaviours and features of dogs in order to assess the circumstances. Nevertheless, because there is no evidence that increased knowledge about dog behaviour reduces the number of dog bites [30], it is likely that multi-factor dog bite prevention programs would be most efficient to reduce the number of such incidents. These programs would, ideally, include: dog training exercises, dog behaviour interpretation training for guardians, dog ownership regulation, strict child and dog supervision rules. If we take studies investigating young children’s awareness of dangerous situations in the home and in road environments as an example, there is evidence that there is the potential for children’s understanding to be enhanced by training about what makes specific situations dangerous. Despite the studies in the latter field suggesting that young children are poor at identifying dangerous situations, these do show that young children have some awareness of danger which can be enhanced by training [26, 31, 32]. They suggest that safety education needs to be aimed at making danger salient to children, as well as teaching them the nature of the danger and strategies for dealing with it.
